# Neurocellular Stress Response to Mojave Type A Rattlesnake Venom: Study of Molecular Mechanisms Using Human iPSC-Derived Neural Stem Cell Model

**DOI:** 10.3390/biom15030381

**Published:** 2025-03-06

**Authors:** Satish Kumar, Miriam Aceves, Jose Granados, Lorena Guerra, Felicia Juarez, Earl Novilla, Ana C. Leandro, Marcelo Leandro, Juan Peralta, Sarah Williams-Blangero, Elda E. Sanchez, Jacob A. Galan, John Blangero, Joanne E. Curran

**Affiliations:** 1 Division of Human Genetics and South Texas Diabetes and Obesity Institute, University of Texas Rio Grande Valley School of Medicine, McAllen, TX 78504, USA; miriam.aceves@utrgv.edu (M.A.); earl.novilla01@utrgv.edu (E.N.); sarah.williams-blangero@utrgv.edu (S.W.-B.); 2 Division of Human Genetics and South Texas Diabetes and Obesity Institute, University of Texas Rio Grande Valley School of Medicine, Brownsville, TX 78520, USAmarcelo.leandro@utrgv.edu (M.L.); jacob.galan@utrgv.edu (J.A.G.); john.blangero@utrgv.edu (J.B.); joanne.curran@utrgv.edu (J.E.C.); 3 National Natural Toxin Research Center (NNTRC), Texas A&M University-Kingsville, Kingsville, TX 78363, USA; elda.sanchez@tamuk.edu

**Keywords:** human iPSCs, NSCs, Mojave rattlesnake venom, neurocellular response, molecular mechanisms

## Abstract

The Mojave rattlesnake venom shows significant geographical variability. The venom of Type A animals primarily contains β-neurotoxin referred to as Mojave Toxin (MTX), which makes bites from this snake particularly feared. We performed a genome-wide transcriptomic analysis of the neurocellular response to Mojave Type A rattlesnake venom using induced pluripotent stem cell-derived neural stem cells to unveil the molecular mechanisms underlying the damage caused by this snake’s envenomation. Our results suggest that snake venom metalloproteases, although having a limited repertoire in Type A venom, facilitate venom spread by digesting the tissue’s extracellular matrix. The MTX, which is composed of heterodimers of basic and acidic phospholipase-A2, co-opts the host arachidonic acid and Ca^2+^ second messenger mechanisms and triggers multiple signaling cascades, such as the activation of MAPKs and NF-κB-regulated proinflammatory genes; the neurotransmitter overload in excitatory synapses leading to a presynaptic blockade of nerve signals; and the upregulation of unfolded protein response (UPR) due to the depletion of Ca^2+^ from the endoplasmic reticulum. The upregulated UPR and the oxidative stress caused by reactive oxygen species generated in cytochromeP4501A1-mediated hydroxylation of arachidonic acid contribute to mitochondrial toxicity. The activation of UPR, mitochondrial toxicity, and oxidative stress synergistically contributed to apoptotic and ferroptotic cell death.

## 1. Introduction

Most venomous snakes in the United States belong to the subfamily Crotalidae (pit vipers), and snake bites are predominantly concentrated in the southern and southwestern states [1,2]. The Mojave rattlesnake (*Crotalus scutulatus scutulatus*) is a highly venomous pit viper. It is found in the arid regions of southeastern California, southern Nevada, the southwestern half of Arizona, the southwestern corner of New Mexico, western Texas, and deep into mainland Mexico [3,4]. Although Mojave rattlesnakes are responsible for relatively fewer snakebite envenomations and deaths in the United States when compared to other species, they are often cited as the most dangerous snake, with an estimated untreated lethality rate as high as 30–40% [1].

The Mojave rattlesnake venom is poorly characterized and shows significant geographical variability in its composition. The venom of Type A animals primarily contains a potent presynaptic β-neurotoxin composed of heterodimers of basic and acidic phospholipase A2 (PLA_2_) subunits, commonly referred to as Mojave Toxin (MTX), but has little hemorrhagic activity. The Type A animals are found in southern California, southern Nevada, southwest Utah, western New Mexico, southwestern Arizona, and southwestern Texas. The venom of Type B animals lacks the MTX but has a more common proteolytic and hemorrhagic activity due to the high expression of snake venom metalloproteases (svMPs). Type B animals are confined to central Arizona. Less frequently than Type A and Type B animals, the venom of some Mojave rattlesnakes found in an intergrade zone between Type A and Type B habitats in central Arizona possesses both MTX and cytotoxic and hemotoxic components and they are designated as Type A+B [5,6]. The subsequent discovery of a specific myotoxin (referred to as Type M) in Mojave rattlesnakes in southeastern Arizona increased the number of Mojave rattlesnake venom phenotypes to six (i.e., A; A+M; B, B+M; A+B; and A+B+M) [7]. A more recent comparative transcriptomic analysis of Mojave rattlesnake venom gland specimens confirmed the dichotomy of Mojave rattlesnake venom. The venom of Type A animals contained significantly fewer toxins, but exclusively expressed neurotoxic MTX, than the venom of Type B animals which contained a diverse repertoire of hemorrhagic C-type lectins (CTLs) and svMPs. The venom of Type A animals contained only two svMPs (svMPIII-2 and svMPIII-3) and different combinations of CTLs regardless of venom type. While there are individual variations in the myotoxins expressed, myotoxin specificity to venom types is still not clear [8]. The myotoxins disrupt sodium channels in muscle cells causing muscle paralysis and lowered median lethal dose (LD_50_) when present. The median lethal dose (LD_50_) for Mojave rattlesnake venom ranges from 0.3 to 4 mg/kg body weight [7]. However, a LD_50_ as low as 0.056 mg/kg of body weight was reported for purified MTX in intravenous LD_50_ tests in mice [9].

The retrospective examination of past Mojave rattlesnake envenomation records has shown stereotypic hemorrhagic and/or neurotoxic symptoms. However, the small sample size, inconclusive snake identification, overlapping neurotoxic symptoms caused by the venoms of other species of rattlesnakes found within the Mojave rattlesnake range, intraspecies variation within Mojave venom, and significant variability in medical intervention limit the understanding and extent of the clinical symptoms of Mojave rattlesnake envenomations [7,10].

The two most consequential classes of toxins in Mojave rattlesnake venom are MTX and svMPs. The neurotoxic effect of MTX is what makes a Mojave rattlesnake bite so feared. As discussed above, MTX is a snake venom phospholipase A2 (svPLA_2_) β-neurotoxin. The svPLA_2s_ are highly active components found in the venoms of Elapid and some Viperid snakes. They are known to have extensive pathological effects and are directly responsible for both early- and late-onset symptoms, as well as having synergistic and regulatory roles for other snake venom components. However, the precise mechanism of svPLA_2s_-induced neuromuscular toxicity and paralysis remains to be fully understood [11,12,13].

To investigate the molecular mechanisms of Mojave rattlesnake venom neurocellular toxicity, we performed a transcriptome-wide analysis of the neurocellular response to Mojave Type A rattlesnake venom challenge in the induced pluripotent stem cell (iPSC)-derived neural stem cells (NSCs) of four individuals of our Mexican American Family Study (MAFS). The human iPSC-derived NSCs, which we have previously shown to be the close surrogate of the human primary dorsal neuroepithelium and are relevant cells for modeling human neurocellular stress response [14,15], were challenged with two concentrations (10 µg/mL and 30 µg/mL, respectively) of Mojave Type A rattlesnake venom in in vitro cultures and then analyzed by genome-wide RNA sequencing both at baseline (vehicle-treated control) and post-venom-challenge at 4 and 24 h time points.

## 2. Materials and Methods

The iPSC reprogramming and NSC differentiation methodology used in this study were described in our previous publications [14,15,16,17].

### 2.1. Snake Venom Challenge

Validated, low-passage NSCs generated from the samples of four different individuals were seeded in Geltrex (Thermo Fisher Scientific, Waltham, MA, USA) -coated tissue culture vessel at a cell density that reached 70–80% confluency in 24 h. During the first 24 h, the NSCs were cultured in NSC expansion medium consisting of 50% Neurobasal medium, 50% Advanced DMEM/F12 medium, and 1X neural induction supplement (all from Thermo Fisher Scientific, Waltham, MA, USA) along with 5 µM ROCK inhibitor Y27632 (ATCC, Manassas, VA, USA). Subsequently, when the cells reached 70–80% confluency, the medium was replaced with NSC expansion medium containing vehicle control (phosphate-buffered saline), 10 µg/mL, and 30 µg/mL pooled Mojave Type A rattlesnake venom (obtained from National Natural Toxins Research Center/NNTRC, Kingsville, USA), respectively. Half of the NSC cultures for each treatment (i.e., vehicle control, 10 µg/mL, and 30 µg/mL venom challenge) were harvested after 4 h, and the other half after 24 h. Both vehicle-treated control NSCs and snake venom-challenged NSCs from each condition were analyzed by genome-wide deep RNA sequencing. Quality controls on generated NSCs were performed by analyzing the differential gene expression between iPSCs and vehicle-treated control NSCs throughout the experiments to confirm that the generated NSCs maintained their desired characteristics.

### 2.2. Intracellular Ca^2+^ Assay

Validated NSCs generated from two individuals were seeded in a Geltrex (Thermo Fisher Scientific, Waltham, MA, USA)-coated 96 well tissue culture plate at 40 thousand cells per well in the NSC expansion medium (described above) supplemented with 5 µM ROCK inhibitor Y27632 (ATCC, Manassas, VA, USA). After 24 h, cells were loaded with 3 µM Fluo-4 AM (Invitrogen™, Thermo Fisher Scientific, Waltham, MA, USA) in the NSC expansion medium supplemented with 0.1% Pluronic F-127 (all from Invitrogen™, Thermo Fisher Scientific, Waltham, MA, USA) for 1 h. After 1 h of incubation with Fluo-4 AM, cells were washed three times with Hanks’ Balanced Salt Solution, and baseline (t_0_) fluorescence was recorded using the Perkin Elmer Operetta high content screening system. The NSCs were then challenged with vehicle control and 10 µg/mL and 30 µg/mL Mojave Type A rattlesnake venom, and fluorescence was recorded at 2 min and with maximum speed thereafter (about every 1.12 min) for a total of 10 time points (total 12.5 min post-challenge). Mean fluorescence intensities were quantified in the image region containing cells using Perkin Elmer Operetta Harmony software (v4.1).

### 2.3. RNA Extraction and Sequencing

The total RNA from vehicle-treated control NSCs and snake venom-challenged NSCs was extracted using the RNeasy Mini Kit (Qiagen, Germantown, MD, USA) and according to the manufacturer’s protocol. The extracted RNA samples were then quantified and assessed for quality using a NanoDrop 2000 Spectrophotometer (Thermo Fisher Scientific, Waltham, MA, USA) and an Agilent 2200 TapeStation system (Agilent, Santa Clara, CA, USA).

RNA sequencing, of vehicle-treated control NSCs and snake venom-challenged NSCs, was performed on an Illumina NovaSeq 6000 instrument using the Illumina Stranded mRNA Prep Ligation Kit. Briefly, mRNA sequencing libraries were prepared from each sample of 1 µg high-quality total RNA using the reagents supplied in Illumina Stranded mRNA sample preparation kit v2 (Illumina, Inc., San Diego, CA, USA). First, oligo-dT magnetic beads (supplied in the kit) were used to enrich poly-A tail containing mRNA molecules from the total RNA. The enriched mRNA was then fragmented into approximately 200–600 base pair-sized molecules using divalent cations and elevated temperature. Next, first- and second-strand cDNA synthesis was performed using reverse transcriptase, random primers, DNA polymerase-I, and RNase H. The synthesized cDNA fragments were then end-repaired, and adaptor ligations were carried out. Lastly, the resulting cDNA libraries were purified, enriched by PCR, and then sequenced on an Illumina NovaSeq 6000 instrument.

### 2.4. RNA Sequencing Analyses

The Illumina bcl2fastq2 software v2.20.0 (Illumina, Inc., San Diego, CA, USA) was used to generate and demultiplex raw fastq sequence files. Following pre-alignment quality checks, the sequences were aligned to the human genome assembly GRCh38 (hg38) and mapped to RefSeq transcripts using StrandNGS software v4.1 (Strand Life Sciences Pvt. Ltd., Bangalore, India). The aligned reads underwent filtering based on default read quality metrics, and log transformation and “DESeq” normalization were applied. Known genes/mRNAs with a normalized read count (NRC) ≥ 10 and conditions as described in the results were considered for various differential gene expression analyses.

### 2.5. Differential Gene Expression Analyses

We comprehensively analyzed gene expression changes in response to the Mojave Type A rattlesnake venom challenge in NSCs. We used one-way ANOVA and expression fold change (FC) analyses to identify genes that were significantly differentially expressed (DE) between the venom-challenged NSCs and the vehicle-treated control NSCs. Specifically, genes with a one-way ANOVA *p*-value ≤ 0.05, and FC absolute (FC abs) ≥ 1.5 between the pair(s) of conditions were considered DE.

As indicated above, we also performed quality control assessments to confirm that the generated NSCs maintained their desired characteristics throughout the experiments by comparing the gene expression between the iPSCs and the NSCs, which served as the vehicle controls. For this comparison, genes with moderated *t* statistics, FDR-corrected *p*-value ≤ 0.05, and FC abs ≥ 2.0 were considered to be DE.

### 2.6. Functional Annotations and Enrichment Analyses

Functional annotations and enrichment analyses of the gene sets of interest, identified by the differential gene expression analyses between iPSC and NSCs for quality control and between vehicle-treated control and venom-challenged NSCs, were performed using the Kyoto Encyclopedia of Genes and Genomes (KEGG) database [18,19,20], Ingenuity Pathway Analysis (IPA) platform (QIAGEN Digital Insights, Redwood City, CA, USA), and ‘Enrichr’ and ‘ShinyGO v0.81′ gene set enrichment analysis web tools [21,22] (accessed November–December 2024). To map gene sets to KEGG pathways maps, KEGG mapper v5 web tool was used. The ’Enrichr’ tool implements several enrichment scores as described by Chen et al. (2013) [21]. We ranked our ‘Enrichr’ results based on computed Fisher exact test *p*-values. In ‘ShinyGO’ enrichment analyses, FDR is calculated based on the nominal *p*-value from the hypergeometric test. For ‘ShinyGO’ enrichment analyses, we used FDR-corrected *p*-values ≤ 0.05 for statistical significance. In IPA, right-tailed Fisher’s exact test FDR-corrected *p*-values were used for enrichment significance, and the direction of functional change was assessed by the activation *z*-score detailed in Kramer et al. (2014) [23]. Briefly, in IPA activation *z*-score is used to infer activation states of an enriched biological function. The basis of this inference is the relationship between genes and biological function(s) that are literature-derived. The direction of effect (up- or down-regulated) is determined by DE genes in the data set and the direction of the gene’s effect on the biological function. A positive *z*-score indicates upregulation and a negative *z*-score indicates downregulation of the biological function.

## 3. Results

### 3.1. iPSC-Derived NSCs

**Figure 1:**
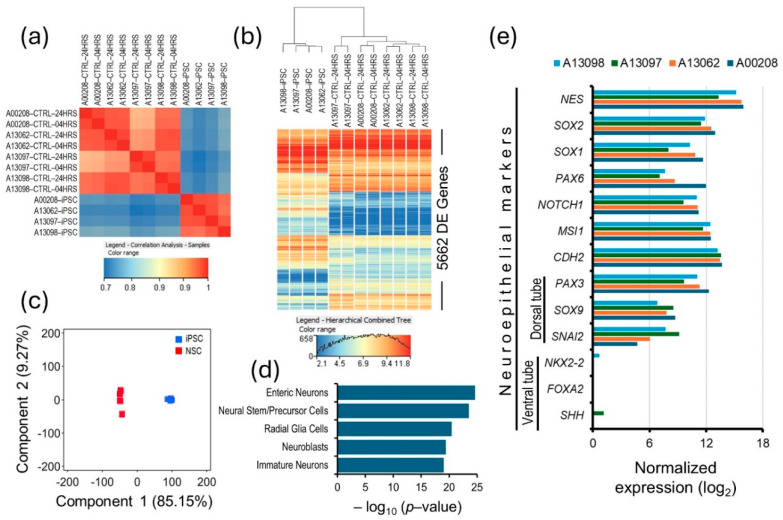
Characterization and quality control of generated NSCs. (**a**) A correlation coefficient (*r*^2^) plot based on the iPSCs and their differentiated NSCs’ expressed transcriptomes (16021 genes with NRC ≥ 10 in iPSCs and/or differentiated NSCs). (**b**) Heat map of transcriptome-wide differentially expressed genes between iPSCs and their differentiated NSCs. (**c**) Principal components plot based on the iPSCs’ and NSCs’ DE transcriptomes. (**d**) Cell type gene set (PanglaoDB Augmented 2021) enrichment of the NSCs’ upregulated transcriptome. (**e**) A bar plot of the neuroepithelial marker gene expression in the generated NSCs.

To confirm that the generated cells were uniform across all samples, possessed transcriptomic and functional characteristics of human NSCs, and maintained such characteristics throughout the experiments, we compared gene expression profiles of iPSCs and the generated NSCs, which were vehicle-treated controls from 4 and 24 h time points. A high correlation between differentiated NSC’s expressed transcriptome, (16,021 genes with NRC ≥ 10 in iPSCs and/or differentiated NSC lines) across all four samples (*r*^2^ [95% CI] = 0.96 ± 0.024), validates a uniform differentiation and similar transcriptomic profiles of the generated NSCs (Figure 1a). A differential gene expression analysis between iPSCs and the differentiated NSCs identified 5662 DE genes, with 2447 being upregulated in the differentiated NSCs. These upregulated genes showed significant enrichment in the PanglaoDB single-cell gene sets of enteric neurons, neural stem/precursor cells, radial glia cells, neuroblasts, and immature neurons. Additionally, principal component analysis of the DE genes shows that about 85% of the differences in gene expression between iPSCs and the generated NSCs were due to the differentiation process (Figure 1b–d). These results suggest a discrete but uniform resetting of the iPSC transcriptome during differentiation, and the changed transcriptome aligns with the characteristics of NSCs. For a more comprehensive characterization, we investigated the expression of neuroepithelial markers in the generated NSCs; a high expression of neuroepithelial genes Nestin (*NES*), Paired box 6 (*PAX6*), SRY-box transcription factors 1 and 2 (*SOX1, SOX2*), Notch receptor 1 (*NOTCH1*), Musashi RNA-binding protein 1 (*MSI1*), and Chromodomain helicase DNA-binding protein 2 (*CHD2*), and of the genes of the dorsal tube neuroepithelium, Paired box 3 (*PAX3*), Growth differentiation factor 7 (*GDF7*), SRY-box transcription factor 9 (*SOX9*), and Snail family transcriptional repressor 2 (*SNAI2*) across all samples validates the dorsal tube neuroepithelial transcriptomic profile of the generated NSCs (Figure 1e). Furthermore, we have previously shown that our iPSC-generated NSCs possessed the apical–basal polarity characteristics of the neuroepithelium [15]. Overall, these results show that our iPSC-generated cells possessed NSC characteristics and maintained such characteristics throughout the snake venom challenge experiments.

### 3.2. Neurocellular Response to the Mojave Type A Rattlesnake Venom

**Figure 2:**
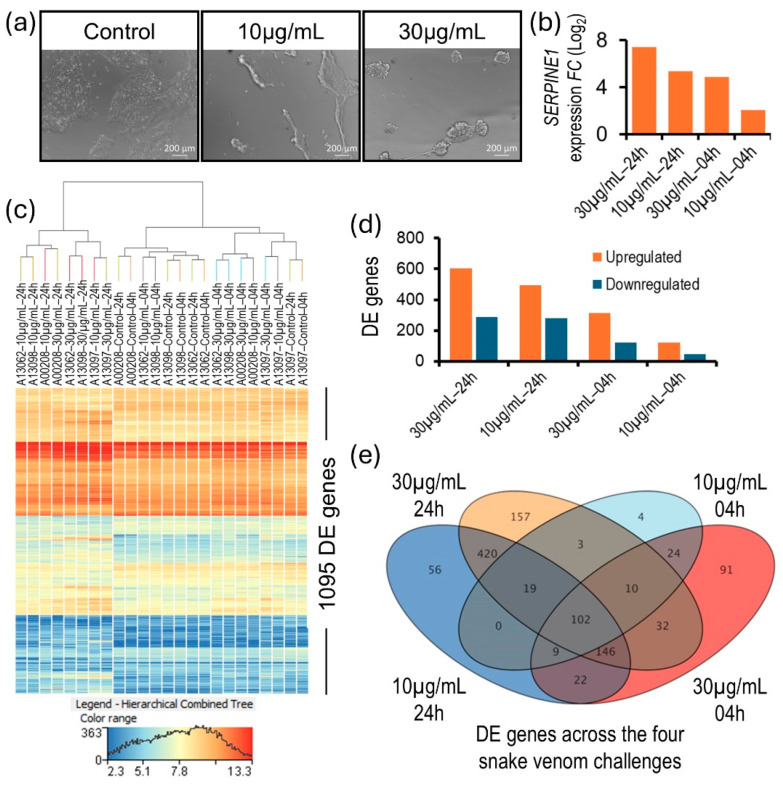
Morphological and transcriptional effect of Mojave Type A rattlesnake venom on NSC culture. (**a**) Brightfield image panel showing the phenotypic effect of venom on NSC culture. (**b**) Time and dose-dependent change in *SERPINE1* expression in NSCs across the four venom challenge conditions. (**c**) Heat map of transcriptome-wide differential gene expression between the vehicle-treated and the venom-challenged NSCs. (**d**) Significantly up- and down-regulated genes in venom-challenged NSCs across the challenge conditions. (**e**) Venn diagram of DE genes across the four snake venom challenge conditions.

The first visible sign of the snake venom challenge on NSC culture was that it digested the basement matrix within an hour and the cells self-organized into 3D suspension structures. Also, the expression of the gene serpin family E member 1 (*SERPINE1*), which encodes a member of the serine proteinase inhibitor superfamily, was among the top upregulated genes (Figure 2a,b). These results suggest a potent protease component in the Mojave Type A rattlesnake venom that causes tissue injury and plausibly accelerates the spread of venom toxins. To investigate the transcriptome-wide neurocellular cellular response to Mojave Type A snake venom, 15,654 genes with an NRC ≥ 10 in at least one of six conditions of the snake venom challenge (i.e., vehicle-treated control and 10 µg/mL and 30 µg/mL snake venom-challenged, both at 4 and 24 h time points, respectively) were considered expressed and included in the genome-wide differential gene expression analysis. A pairwise differential gene expression analysis between vehicle-treated controls and 10 µg/mL and 30 µg/mL Mojave Type A rattlesnake venom-challenged NSCs at time points of 4 and 24 h identified 1095 genes that were significantly (one-way ANOVA *p*-value ≤ 0.05; FC *abs* ≥ 1.5) DE (Table S1 and Figure 2c). While time showed an augmenting effect, a higher dose of snake venom (30 µg/mL) accelerated the neurocellular response. The 30 µg/mL snake venom challenge for 24 h resulted in the most with 889 DE genes and higher cell death, followed by 774 DE genes in the 10 µg/mL venom challenge for 24 h, 436 DE genes in the 30 µg/mL venom challenge for 4 h, and 171 DE genes in the 10 µg/mL venom challenge for 4 h (Figure 2d,e).

**Figure 3:**
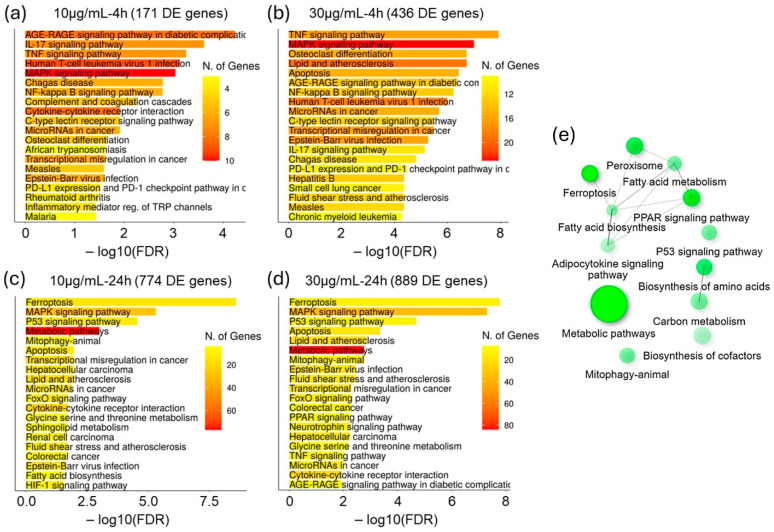
DE gene sets enrichment in KEGG pathways. (**a**–**d**) Top 20 enriched KEGG pathways in genes that were DE post–10 µg/mL for 4 h, –30 µg/mL for 4 h, –10 µg/mL for 24 h, and –30 µg/mL for 24 h Mojave Type A rattlesnake venom challenges, respectively. (**e**) Network of top 20 KEGG pathways that were enriched in 633 DE genes, which were specific to the NSC’s response to the longer 24-h venom challenge. Two pathways (nodes) are connected if they share 20% or more genes. Darker nodes are more significantly enriched gene sets. Bigger nodes represent larger gene sets. Thicker edges represent more overlapped genes.

The enrichment analysis of the NSCs’ transcriptomic response to the Mojave Type A rattlesnake venom challenge suggests an initial proinflammatory insult that progressed into a broad multipronged cell death and degenerative process (Figure 3). The genes that were DE in the shorter (4 h) challenge showed significant enrichment in mitogen-activated protein kinase (MAPK) and nuclear factor-kappa B (NF-κB) signaling regulated proinflammatory pathways (Figure 3a,b). The longer (24 h) duration of the venom challenge, however, elicited a broader response, with a significant increase in the number of DE genes and a sundry of enriched functional pathways. While MAPK and the proinflammatory pathways continued to be highly enriched, ferroptosis, p53 signaling, apoptosis, and mitophagy pathways were among the top enriched in the NSC’s response to the 24-h venom challenge. Additionally, pathways associated with oxidative stress and fatty acid metabolism were among the top 20 enriched pathways (Figure 3c,d).

Interestingly, the 633 DE genes that were specific to the longer 24-h venom challenge (Figure 2e) response, were enriched in the pathways suggestive of the oxidative stress- and lipid peroxidation-associated cell death program/degenerative process (Figure 3e). For a more in-depth understanding of the human neurocellular response mechanisms to the Mojave Type A rattlesnake venom, we focused our investigation on key pathways from the above enrichment results.

### 3.3. MTX Co-Opts Host Arachidonic Acid and Ca^2+^ Signaling in Rapidly Amplifying Insults

**Figure 4:**
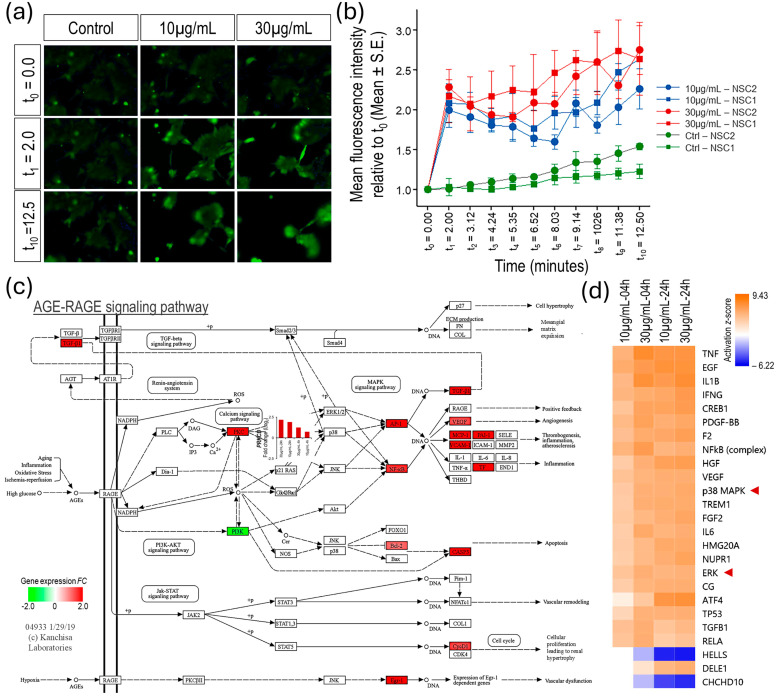
MTX upregulates MAPK and proinflammatory gene program. (**a**) Representative image panel showing increased intracellular Ca^2+^ (measured using relative fluorescence intensity of cell-permeable calcium indicator Fluo-4 AM) in the venom-challenged NSCs. (**b**) Relative mean fluorescence intensity (t*_i_*/t_0_) of calcium indicator Fluo-4 AM between vehicle controls and post-venom-challenged NSCs (t_0_ = mean fluorescence intensity pre-challenge, t*_i_* = mean fluorescence intensity at time points post-challenge). (**c**) KEGG AGE-RAGE signaling pathway map showing activation of PKC, MAPKs, and NF-κB regulated proinflammatory gene program in venom challenged NSCs. (**d**) Upstream regulators that were significantly enriched and predicted to be highly up- or down-regulated (activation z-score-absolute ≥ 5.0) in post-venom-challenged NSCs.

Mapping genes, which were DE across the four venom challenges to enriched KEGG pathways suggests a significant role of intracellular Ca^2+^ in the neurocellular damage caused by Mojave Type A rattlesnake venom. The svPLA_2s_ hydrolyze glycerol–easter bonds of glycerophospholipids in the cell membrane releasing fatty acids metabolites, largely arachidonic acid, and lysophospholipids [24]. The disruption/permeabilization of the cell membrane by MTX (a svPLA_2_) catabolism and the action of metabolites, arachidonic acid, and lysophospholipids likely leads to the influx of Ca^2+^ into the cytosol from extracellular space and the intracellular stores. The intracellular Ca^2+^ flux measured in triplicate in two different NSC samples using cell-permeant Fluo-4 AM calcium indicator and high-content screening/imaging analysis showed a rapid and sustained increase in intracellular Ca^2+^ post-venom challenge (Figure 4a,b). The NSCs’ transcriptomic response to the venom challenge also suggests that the released arachidonic acid and intracellular Ca^2+^ overload activates multiple signaling pathways including (1) the activation of protein kinase C (PKC), MAPKs, and S100 proteins resulting in an upregulated NF-κB proinflammatory gene program, which constitutes the venom’s proinflammatory damage; (2) neurotransmitter overload by increased vesicle fusion leading to prey paralysis; and (3) the activation of endoplasmic reticulum (ER) stress-induced upregulation of unfolded protein response (UPR) leading to apoptosis and upregulated neurodegenerative processes.

The DE genes mapped to the enriched KEGG AGE–RAGE signaling and MAPK signaling pathways and the repertoire of upstream regulators predicted to be highly activated (activation z-score ≥ 5.0) in DE genes suggests that the venom-challenge-activated MAPKs and the expression of its downstream NF-κB regulated proinflammatory genes by activating PKC. While classical PKC isoforms (PKC-α, PKC-β_I_, PKC-β_II_, and PKC-γ) that are activated by Ca^2+^ and diacylglycerol (DAG) did not show significant activation. Intriguingly, the PKC-δ mRNA expression (*PRKCD FC*-range = 1.65 to 4.5) and predicted activation *z*-score (*z*-score range = 2.4 to 3.8) showed a dose and time-dependent upregulation in the venom-challenged NSCs (Figure 4c,d, Figure S1, and Table S1). PKC-δ lacks the calcium-binding domain and its activation is dependent on diacylglycerol (DAG), a second messenger signaling lipid that also stimulates Ca^2+^ release [25]. Importantly, the activity of the phospholipase C (PLC) enzyme is required for the production of DAG [26]. Additionally, positive feedback loops in the MAPK signaling cascade also seem to play a role in the sustained proinflammatory damage (Figure S1).

Since the increase in intracellular Ca^2+^ triggers cellular depolarization and neurotransmitter release, it has been suggested that intracellular Ca^2+^ may have a role in the MTX presynaptic blockade caused by neurotransmitter overload [27].

**Figure 5:**
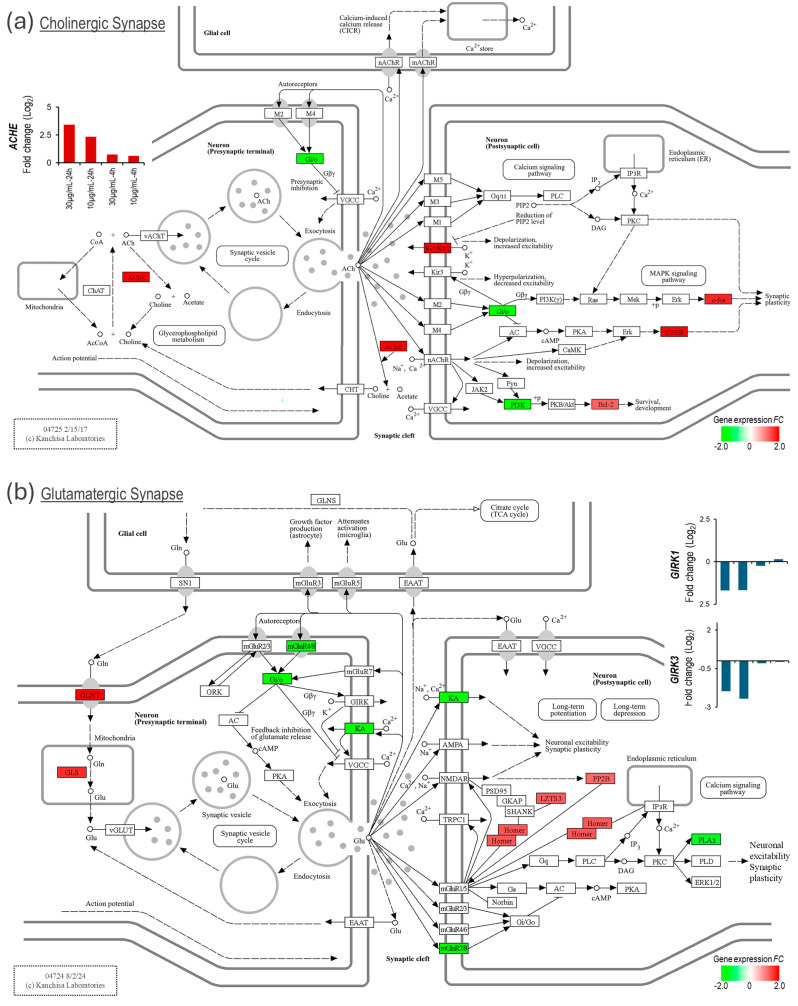
Mojave Type A rattlesnake venom-induced expression change in excitatory synapse genes. (**a**) KEGG Cholinergic synapse map showing upregulated *ACHE* expression in venom-challenged NSCs. (**b**) KEGG Glutamatergic synapse map showing altered expression of ionotropic and metabotropic glutamate receptors in venom-challenged NSCs.

Therefore, next, we investigated the excitatory cholinergic and glutamatergic synapses for these plausible events and their transcriptomic signature (Figure 5a,b). Interestingly, the expression of the *ACHE* gene, which encodes acetylcholinesterase that degrades acetylcholine, was significantly upregulated (˃10-fold increase in NSCs challenged with 30 µg/mL venom for 24 h). In addition, the expression of genes *GRIK1* and *GRIK3*, which encode proteins belonging to kainate ionotropic glutamate receptors (KA-iGluRs), was significantly downregulated (Figure 5b). KA-iGluRs are activated by glutamate and are responsible for fast excitatory neurotransmission. These transcriptomic changes in the venom-challenged NSCs indicate responses to neurotransmitter overload in the excitatory synapses.

**Figure 6:**
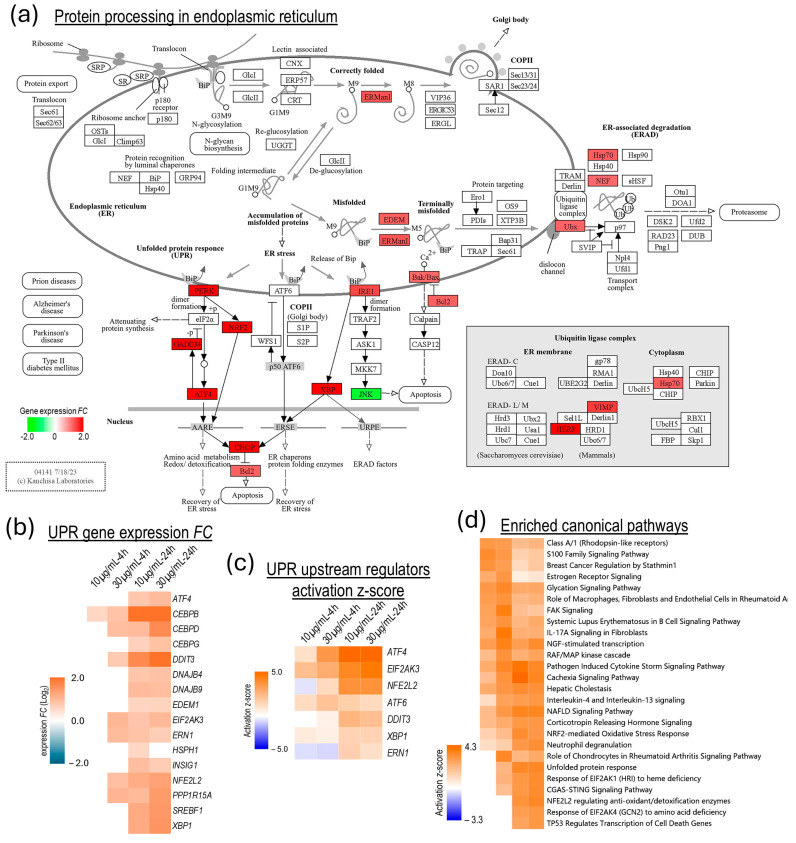
Unfolded protein response in venom-challenged NSCs. (**a**) KEGG pathway map of protein processing in ER showing upregulated UPR-associated genes. (**b**) Expression heat map of UPR-associated genes across the venom challenge conditions. (**c**) Activation *z*-score heat map of key UPR-associated transcription factors. (**d**) Heat map of significantly enriched and highly activated (activation *z*-score ≥ 3.0) IPA canonical pathways.

The third apparent effect of the altered Ca^2+^ homeostasis in venom-challenged NSCs, which plausibly contributes to the venom-induced cell death and degenerative processes, was the upregulation of ER stress-induced UPR pathways (Figure 6).

Genes involved in the ER-associated degradation (ERAD) of misfolded proteins and two of the three UPR pathways (PERK/EIF2AK3 and IRE1/ERN1) were significantly upregulated in the venom-challenged NSCs, albeit in a time-dependent manner (Figure 6a,b). Intriguingly, the activation z-score for the eukaryotic translation initiation factor 2-alpha kinase 3 (EIF2AK3; also known as PERK) pathway, including EIF2AK3 itself, and the nuclear factor erythroid-derived 2-like 2 (NFE2L2; also known as NRF2), and activating transcription factor 4 (ATF4) transcription factors, was significantly higher than the endoplasmic reticulum to nucleus signaling 1 and X-box binding protein 1 (ERN1-XBP1) pathway, suggesting that EIF2AK3, NFE2L2, and ATF4 may also have a synergistic relationship with other venom-induced cellular pathways/processes (Figure 6c). NFE2L2 and its regulated genes are also activated by oxidative stress. Eukaryotic translation initiation factor 2 alpha kinase 1 (EIF2AK1) is activated by heme deficiency and oxidative stress, and eukaryotic translation initiation factor 2 alpha kinase 4 (EIF2AK4) responds to amino acid deficiency. These integrated stress response kinases (EIF2AK1, EIF2AK3, and EIF2AK4) phosphorylate eukaryotic translation initiation factor 2 subunit alpha (EIF2S1; also referred to as eIF2α), which sequester cellular translation but activates translation of certain transcription factors, such as ATF4, activating transcription factor 5 (ATF5), and DNA damage-inducible transcript 3 (DDIT3; also referred to as CHOP). ATF4 upregulates gene programs that ameliorate cellular stress to restore ER homeostasis, redox homeostasis, and mitochondrial function. Unresolved stress, however, leads to DDIT3/CHOP-mediated apoptosis (Figure 6d).

### 3.4. Mojave Type A Rattlesnake Venom Induces Mitochondrial Toxicity in NSCs

**Figure 7:**
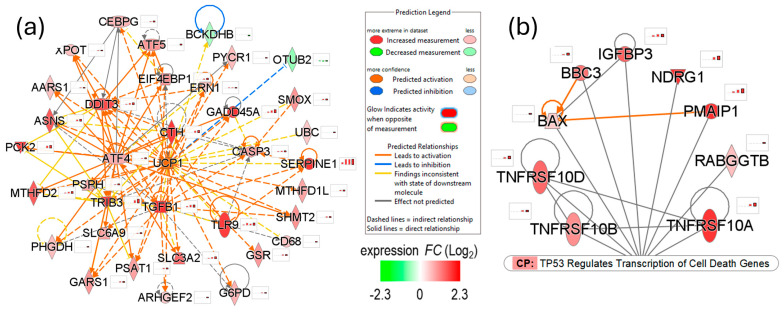
Mojave Type A rattlesnake venom-induced mitochondrial toxicity in NSCs. (**a**) Gene network showing predicted upregulation of UCP1, suggesting mitochondrial energy decoupling. (**b**) Network of TP53-regulated cell death genes indicating mitochondrial membrane permeabilization and upregulation of both intrinsic and extrinsic programmed cell death in venom-challenged NSCs.

Previous studies have reported that the PLA_2_ enzymatic activity of venom β-neurotoxins, as well as even a low concentration of its metabolite ‘arachidonic acid’, induces mitochondrial permeability transition in neurons, which causes mitochondrial energy decoupling and Ca^2+^ release by mechanism(s) that are independent of mitochondrial uncoupling protein 1 (UCP1) [28,29]. The upstream regulator analysis of the genes that were DE post-venom challenge in NSCs showed a significant predicted upregulation of the UCP1 (UCP1 activation *z*-score ≥ 3.0 in NSCs venom challenged for 24 h); however, its mRNA expression was below the detection threshold and did not change post-venom challenge, suggesting a similar mitochondrial permeability transition in venom-challenged NSCs as reported previously in neurons. Additionally, significant upregulation of TP53-transcriptional targets, BCL2-associated X protein (*BAX*), phorbol-12-myristate-13-acetate-induced protein 1 (*PMAIP1*; also known as *NOXA*), and BCL2 binding component 3 (*BBC3*; also known as *PUMA*), in venom-challenged NSCs also suggests mitochondrial membrane permeabilization and cytochrome C release (Figure 7a,b).

### 3.5. Arachidonic Acid Metabolism Induces Oxidative Stress in Venom-Challenged NSCs

**Figure 8:**
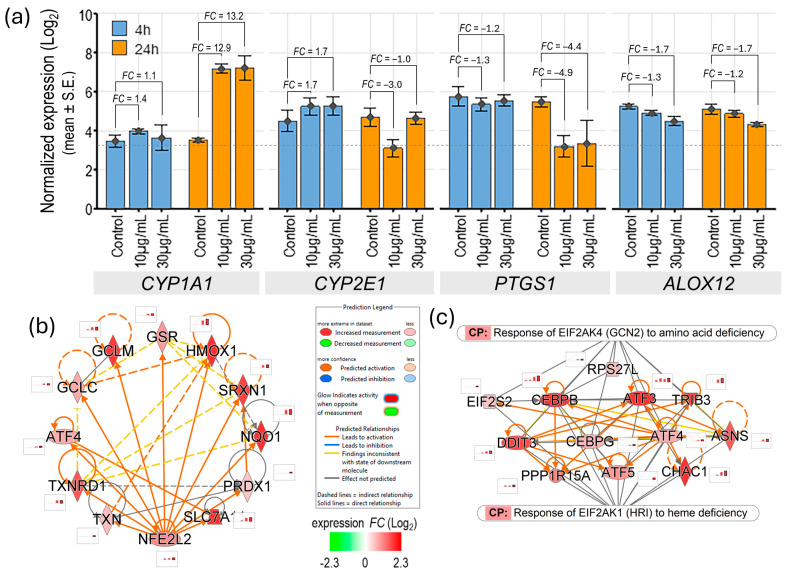
Arachidonic metabolism is associated with oxidative stress in venom-challenged NSCs. (**a**) Gene expression profiles of arachidonic acid metabolizing enzymes in control and venom-challenged NSCs. (**b**) Gene network showing interaction map and gene expression profiles of NFE2L2/NRF2-regulated anti-oxidant/detoxification enzymes in venom-challenged NSCs. (**c**) Gene networks showing activation of integrated stress response kinases gene programs in venom-challenged NSCs.

In human cells, free arachidonic acid is metabolized by three enzyme systems, the cyclooxygenases (COXs, also referred to as PGG/H synthases), lipoxygenases (LOXs), and cytochrome P450 (CYP) enzymes (ω-hydroxylases and epoxygenases), to generate a spectrum of biologically active fatty acid mediators, which act as local hormones and signaling molecules [30]. Interestingly, in NSCs arachidonic acid generated by the MTX activity appears to be predominantly metabolized by the cytochrome P450 1A1 (CYP1A1) enzyme, albeit with some delayed kinetics. The expression of *CYP1A1* in NSCs challenged with 30 µg/mL and 10 µg/mL venom for 24 h was upregulated 13.2- and 12.9-fold, respectively. However, its expression changed nominally during the shorter 4-h venom challenge. The expression of another CYP enzyme gene, *CYP2E1,* showed nominal upregulation~1.7-fold during the shorter 4-h venom challenge but then downregulated during the longer 24-h challenge. Additionally, the expression of prostaglandin-endoperoxide synthase 1 (*PTGS1*; also known as *COX1*) and arachidonate 12-lipoxygenase (*ALOX12*), the only COXs and LOXs enzyme genes that were expressed in NSCs (NRC ≥ 10 in samples of at least one of the six venom challenge conditions), was significantly downregulated post-venom challenge (Figure 8a).

Human CYP1A1 catalyzes subterminal (ω-n)-hydroxylation of arachidonic acid mainly to 16–19 hydroxyeicosatetraenoic acids (HETEs), which accounts for about 75–90% of the total hydroxylation products, and to a lesser extent to epoxyeicosatrienoic acids (EETs; mainly 14, 15 EETs), about 5–7% of total metabolites [31,32]. These arachidonic acid hydroxylation products (16–19 HETEs and 14,15 EETs) are reported to perform diverse tissue-specific physiological functions, prominently acting as vasodilators, vasoprotective, anti-inflammatory, and inhibitors of voltage-gated Ca^2+^ channels, and ATPase activity [33].

The generation/leakage of reactive oxygen species (ROS) during arachidonic acid hydroxylation by CYP1A1 leads to oxidative stress as indicated by the significant enrichment and predicted activation of the NFE2L2/NRF2-mediated oxidative stress response, as well as the integrated stress response kinase (EIF2AK1 and EIF2AK4) pathways (Figure 8b,c).

### 3.6. Cellular Death and Degeneration in Venom-Challenged NSCs

The enrichment analysis of genes that were DE in venom-challenged NSCs, particularly in the longer 24-h challenge, into KEGG and IPA canonical pathways suggests the activation of ferroptotic and apoptotic cell death by a multipronged cellular response to proinflammatory stress, ER stress, oxidative stress, and mitochondrial toxicity (Figure 3c,d, and Figure 6d).

Both extrinsic and intrinsic apoptotic pathways—marked by the significantly upregulated expression of TRAILR (death receptor) genes, TNF receptor superfamily members 10a, 10b, and 10d (*TNFRSF10A*, *TNFRSF10B*, and *TNFRSF10D*), and *BAX, PMAIP1 (NOXA),* and *BBC3 (PUMA)* genes, respectively—were significantly activated (Figure 7b). The expression of apoptosis effector caspase 3 (*CASP3*) was also significantly upregulated. The upregulated expression of intrinsic pathway genes (*BAX, PMAIP1,* and *BBC3*) leads to mitochondrial membrane permeability and cytochrome C release, which is a central event in apoptotic cell death.

Ferroptosis is a ROS-dependent programmed cell death caused by redox imbalance and involves iron accumulation and lipid peroxidation. As discussed above (Figure 8b), the oxidation of arachidonic acid by CYP1A1 generated high levels of ROS in venom-challenged NSCs. Though the high levels of ROS induced a robust NFE2L2-regulated oxidative stress response in NSCs, it plausibly causes oxidative damage to proteins, lipids, and nucleic acids, including lipid peroxidation, which is a key component of ferroptotic cell death. Additionally, the expression of the Acyl-CoA synthetase long-chain family member 5 (*ACSL5*) gene, which increases ROS production, lipid peroxidation, and induces ferroptosis, was upregulated 6.1- and 3.2-fold in NSCs challenged with 30 µg/mL and 10 µg/mL venom, respectively, for 24 h [34]. The venom-challenged NSCs also showed the upregulated expression of iron uptake, metabolism, and storage genes, transferrin receptor 1 (*TFRC*), heme oxygenase 1 (*HMOX1*), ferritin heavy chain 1 (*FTH1*), and ferritin light chain (*FTL*). While *HMOX1, FTH1,* and *FTL* functions limit iron toxicity their expression is upregulated in ferroptosis [35]. Excessive HMOX1 activity is also shown to cause non-canonical ferroptosis [36]. Interestingly, the expression of anti-ferroptosis glutathione metabolism pathway genes, solute carrier family 7 member 11 (*SLC7A11*), solute carrier family 3 member 2 (*SLC3A2*), glutamate-cysteine ligase catalytic subunit (*GCLC*), and glutamate-cysteine ligase modifier subunit (*GCLM*), was also upregulated in venom-challenged NSCs, suggesting a balancing mechanism between pro-ferroptosis and anti-ferroptosis processes that eventually determine cell fate.

## 4. Discussion

Although not fully characterized, the Mojave Type A rattlesnake venom primarily contains the β-neurotoxin MTX, which is responsible for the neurotoxic effects that make a Mojave rattlesnake bite so feared. Studies thus far have shown that the β-neurotoxin from different snake species produced similar but complex effects by mechanisms that are not fully understood. We employed a genome-wide transcriptomic approach to unveil the mechanisms of Mojave Type A rattlesnake venom neurocellular effect using iPSC-derived human NSCs. We have previously shown that human iPSC-derived tissue-specific cells including NSCs are akin to their primary counterparts and are relevant cell models for studying human pathophysiology and cellular stress response mechanisms [14,15,37,38,39].

The rapid digestion of the basement matrix in venom-challenged NSC cultures and the significant upregulation of *SERPINE1* expression suggest a potent protease component to the Mojave Type A rattlesnake venom (Figure 2a,b). The protease activity of the Mojave Type A rattlesnake venom is likely due to svMPs (though limited to a few types), which induce tissue injury and accelerate the spread of more potent MTX. This is consistent with the general ubiquitous property of venoms, that they rely on barrier penetration rather than specific transport mechanisms to leave the circulation [11,40,41].

At the transcriptome level, the Mojave Type A rattlesnake venom challenge elicited a strong response in NSCs, which started with the activation of MAPK and NF-κB, signaling regulated proinflammatory insult and the dysregulation of neurotransmitter homeostasis in cholinergic and glutamatergic excitatory synapses and then progressed into multipronged cell death and degenerative process with ferroptosis, p53 signaling, apoptosis, and mitophagy among the top enriched pathways in the 24-h venom challenge response (Figure 2 and Figure 3). An in-depth analysis of pathways that were significantly enriched in the NSCs’ response to the venom challenge implicated MTX’s PLA_2_ activity. Although, our data is limited in elucidating MTX specificity to cell surface receptors or cell types. The MTX’s PLA_2_ enzymatic activity, which hydrolyzes membrane glycerophospholipids and generates arachidonic acid and lysophospholipids and plausibly permeabilizes and disrupts the cell membrane integrity, is evident in the rapid influx of intracellular Ca^2+^ (Figure 4a,b). While the influx of Ca^2+^ from extracellular space due to cell membrane disruption by svPLA_2s_ has been suggested, arachidonic acid and lysophospholipids have also been shown to mobilize Ca^2+^ influx into the cytosol by both releasing from intracellular stores and the influx from extracellular space through a wide repertoire of mechanisms [42,43,44,45,46]. Though we did not evaluate the pathways responsible for the influx of intracellular Ca^2+^ in venom-challenged NSCs, the arachidonic acid-mediated release of Ca^2+^ from intracellular stores through inositol 1,4,5-trisphosphate receptors (IP3Rs) and two-pore channels (TPCs) and the influx from extracellular space through transient receptor potential vanilloid type 4 (TRPV4) have been reported in endothelial cells [47,48]. The upregulated expression of the inositol 1,4,5-trisphosphate receptor-interacting protein (*ITPRIP*) gene, which enhances the sensitivity of IP3Rs to intracellular calcium, and the genes associated with ER stress-induced UPR suggest similar pathways of Ca^2+^ release were activated in venom-challenged NSCs.

The venom-induced alteration in fatty acid metabolism and the rapid influx of intracellular Ca^2+^ activated multiple signaling cascades. The activation of MAPKs and NF-κB-regulated proinflammatory cascades were the top enriched pathways in the shorter 4-h NSC response to venom challenge (Figure 2). Our data suggest a significant role of upregulated PKC-δ in the phosphorylation of MAPKs and the overall activation of the proinflammatory cascade in venom-challenged NSCs (Figure 4c,d). PKC-δ lacks the calcium-binding domain and is activated by DAG [49]. DAG also stimulates Ca^2+^ release [25]. However, the mechanism by which DAG is recruited and what role the elevated intracellular Ca^2+^ plays in the activation of MAPKs needs further investigation. Previous studies have shown that arachidonic acid and other fatty acids can activate all three (p38 MAPK, extracellular signal-regulated kinase or ERK, and c-Jun NH_2_-terminal kinase or JNK) branches of the MAPK signaling cascade directly [50]. Additionally, it has also been suggested that svPLA_2s_ are potentially able to co-activate endogenous cellular phospholipases [11]. The MAPK-NF-κB proinflammatory cascades are amplified and sustained through the dose- and time-dependent upregulation of the PKC-δ transcription and the activation of positive feedback loops by the generated cytokines in venom-challenged NSCs (Figure 4c and Figure S1). We did not see a significant contribution of the arachidonic acid metabolites prostaglandins and leukotrienes to the inflammatory process in venom-challenged NSCs, as suggested in some previous reports [11,51,52,53]. The expression of *PTGS1* and *ALOX12*, the only COXs and LOXs enzyme genes expressed in NSCs, was significantly downregulated post-venom challenge (Figure 8a).

Our data suggest that a neurotransmitter overload in the cholinergic and glutamatergic excitatory synapses caused by the venom-induced rapid increase in intracellular Ca^2+^ and the cellular response to mitigate such overload likely has a role in the MTX-induced presynaptic blockade of nerve signals. While the expression of the acetylcholinesterase gene (*ACHE*), which degrades acetylcholine, was significantly upregulated, the expression of the *GRIK1* and *GRIK3* genes, which encode KA-iGluRs proteins, was significantly downregulated in venom-challenged NSCs (Figure 5). These transcriptomic changes in genes playing a critical role in synaptic signal transmission are plausibly a response to neurotransmitter overload in the cholinergic and glutamatergic synapses, respectively, or are caused by mechanisms that are not known. However, they are inhibitory to the postsynaptic transmission of the nerve signal. The involvement of cholinergic and glutamatergic excitatory synapses in these mechanisms suggests that svPLA_2s_ may impact both peripheral and central nervous system signal transmissions.

The activation of UPR, mitochondrial toxicity, and oxidative stress, constitute the degenerative phase of the venom challenge in NSCs and synergistically contribute to cell death by apoptosis and ferroptosis (Figure 3, Figure 6, Figure 7 and Figure 8). To the best of our knowledge, our study, for the first time, shows that the MTX/svPLA_2_-mediated dysregulation of Ca^2+^ homeostasis causes ER stress and the upregulation of the UPR in venom-challenged NSCs (Figure 6). While both the PERK/EIF2AK3 and IRE1/ERN1 UPR pathways were significantly upregulated due to ER stress in the venom-challenged NSCs. Other venom-induced cellular stresses, such as oxidative stress due to the ROS produced in the CYP1A1-mediated oxidation of arachidonic acid and proinflammatory stress, likely have a role in activating other stress response kinases [54]. The EIF2AK1, which is activated by heme deficiency and oxidative stress, and EIF2AK4, which responds to amino acid deficiency, were both predicted to be significantly activated in venom-challenged NSCs (Figure 6d). The activation of integrated stress response kinases (EIF2AK1, EIF2AK3, and EIF2AK4), phosphorylates EIF2S1 (eIF2α), and phosphorylated EIF2S1 sequesters overall protein synthesis but activates the translation of ATF4, PPP1R15A/GADD34, ATF5, and DDIT3/CHOP transcription factors [55,56,57,58,59,60]. ATF4, depending upon its dimerization partner, regulates the transcription of genes that ameliorate cellular stress to restore ER homeostasis, redox homeostasis, and mitochondrial function [61]. However, in unresolved stress, ATF4 can heterodimerize with DDIT3/CHOP and upregulate the expression of apoptotic genes [62,63]. The repertoire of ATF4- and DDIT3-regulated genes in venom-challenged NSCs showed the significant activation of both extrinsic and intrinsic apoptosis pathways (Figure 7b). The ATF4- and DDIT3-mediated transcription of intrinsic apoptosis pathway genes, *BAX, PMAIP1,* and *BBC3,* suggests mitochondrial membrane permeabilization and cytochrome C release in venom-challenged NSCs, which is a central event in apoptotic cell death. Ferroptosis, on the other hand, is driven by ROS production and redox imbalance and involves iron-dependent lipid peroxidation [64]. Ferroptosis seems to be a byproduct of svPLA_2s_‘ fatty acid metabolic cascade and is caused by an imbalance in phospholipid peroxide production and the scavenging capacity of the cell. The high levels of polyunsaturated fatty acid produced by svPLA2 activity [65], high levels of ROS generated in arachidonic acid oxidation by CYP1A1 [66], high levels of free iron from mitochondrial membrane permeabilization, and the influx from extracellular spaces [34], are all conducive to increased phospholipid peroxidation and ferroptosis. The upregulated expression of genes associated with pro-ferroptosis processes, such as lipid peroxidation (*ACSL5*), iron uptake (*TFRC*), iron metabolism (*HMOX1*)*,* and iron storage (*FTH1,* and *FTL*), suggests the induction of ferroptotic cell death in venom-challenged NSCs [35,67]. Interestingly, the glutathione metabolism genes (*SLC7A11*, *SLC3A2, GCLC,* and *GCLM*), which are upstream of the glutathione peroxidase-4 (*GPX4*), the only mammalian enzyme known to catalyze the reduction of phospholipid peroxides, were also significantly upregulated. Although *GPX4* is expressed at high levels in NSCs, its expression did not change post-venom challenge.

## 5. Conclusions

Overall, our study provides a comprehensive repertory of molecular mechanisms involved in the neurocellular damage caused by Mojave Type A rattlesnake venom. We report an extensive list of 1095 genes, which constituted NSCs’ response to the venom challenge across 10 µg/mL and 30 µg/mL venom doses, and 4 and 24 h exposure time points. Our results suggest that svMPs, although having a limited repertoire in Type A venom, facilitate venom spread by digesting tissue’s extracellular matrix; the MTX (β-neurotoxin) co-opts the host arachidonic acid and Ca^2+^ second messenger mechanisms in dose- and time-dependent escalating venom damage. The plausible release of arachidonic acid and the rapid increase in intracellular Ca^2+^ caused by the PLA_2_ activity of MTX trigger multiple signaling cascades and are the initiating step of the overall damage. The activation of MAPK–NF-κB-mediated proinflammatory cascade and neurotransmitter overload in cholinergic and glutamatergic excitatory synapses due to increased intracellular Ca^2+^, as suggested by the gene expression measures, constitute the venom’s initial insult, likely resulting in severe pain, inflammatory damage, and paralysis. Our results also show that PKC-δ, whose expression is significantly upregulated post-venom challenge, has a significant role in activating MAPKs and their downstream proinflammatory cascade. We also report that the MTX/svPLA_2_-mediated dysregulation of Ca^2+^ homeostasis causes ER stress and the upregulation of UPR. The venom challenge also caused mitochondrial energy decoupling that was independent of UCP1 and the permeabilization of mitochondrial outermembrane. Furthermore, our results also suggest that arachidonic acid generated by MTX PLA_2_ activity is predominantly metabolized by the CYP1A1 enzyme into subterminal 16-19 HETEs. The CYP1A1-mediated hydroxylation of arachidonic acid is likely the source of ROS and resulting oxidative stress in venom-challenged NSCs. The activation of UPR, mitochondrial toxicity, and oxidative stress synergistically contribute to the programmed cell death and plausibly constitute the final degenerative phase of the venom-induced damage.

There are a few caveats that should be taken into account while interpreting the results of our study. We studied venom’s neurocellular stress response in iPSC-derived in vitro cultures of human NSCs, which are a relevant cell model to study neurocellular stress response and are close surrogates for primary neural stem/progenitor cells; however, other cell types in the body may confound the response seen in in vitro culture. We mainly focused on mechanisms of venom-induced neurocellular damage evident through transcriptional changes. Lastly, our analysis and interpretation of transcriptional changes relied on existing gene function information in KEGG, IPA, and Gene Ontology databases and are to be experimentally validated.

## Data Availability

The mRNA sequence data generated from the vehicle-treated control and snake venom-challenged NSC lines (n = 24) were submitted to the gene expression omnibus (GEO) archive and are available under the accession number GSE287744.

## Supplementary Materials

The following supporting information can be downloaded at: https://www.mdpi.com/article/10.3390/biom15030381/s1, Table S1: Genes that were significantly differentially expressed (DE) between vehicle-treated controls and Mojave Type A rattlesnake venom-challenged NSCs. Figure S1: MAPK signaling KEGG pathway maps showing mapped genes that were DE at 4 h and 24 h Mojave Type A rattlesnake venom challenge time points.
