# Supplementary material for: Neurocellular Stress Response to Mojave Type A Rattlesnake Venom: Study of Molecular Mechanisms Using Human iPSC-Derived Neural Stem Cell Model
Source: Biomolecules. 2025 Mar 6;15(3):381. doi: 10.3390/biom15030381 (PMC11940042; doi:10.3390/biom15030381)
Supplement: Supplementary file 1 [file biomolecules-15-00381-s001.zip › Figure S1.pdf]

(a) MAPK Signaling (post 4 hours venom challenge)

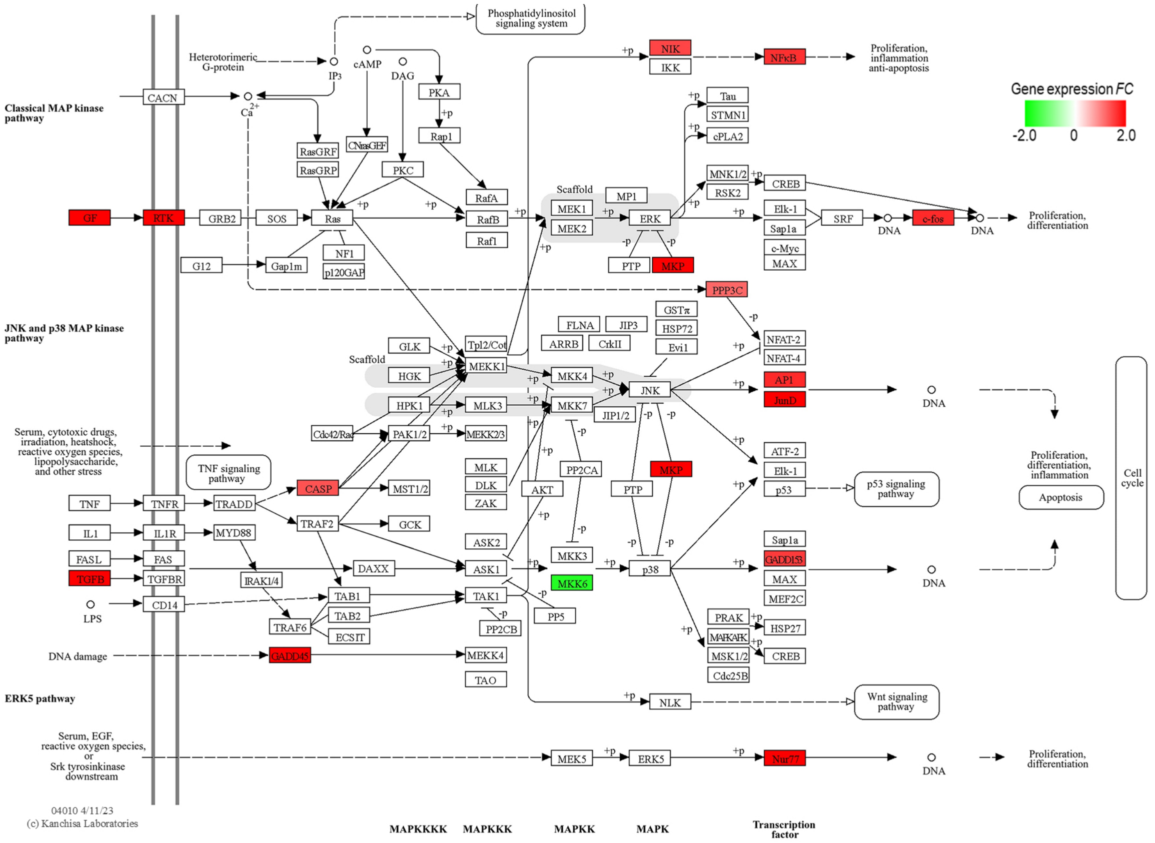

(b) MAPK Signaling (post 24 hours venom challenge)

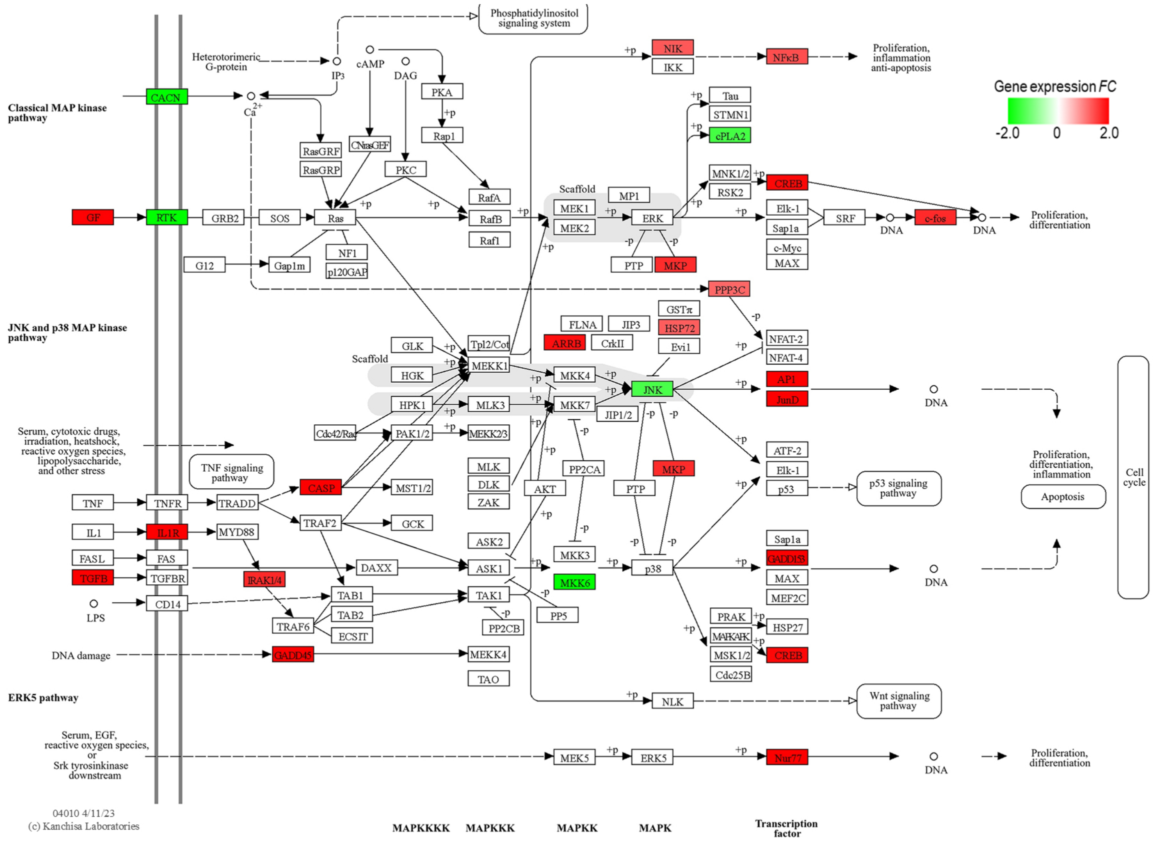

Figure S1: KEGG MAPK signaling pathways maps showing enriched DE genes. (a) Genes that were DE post 4 hours of venom challenge. (b) Genes that were DE post 24 hours of venom challenge.
